# Annual acknowledgement of manuscript reviewers

**DOI:** 10.1186/s13020-015-0034-0

**Published:** 2015-05-16

**Authors:** Siu-wai Leung

**Affiliations:** State Key Laboratory of Quality Research in Chinese Medicine, Institute of Chinese Medical Sciences, Macao, China

## Abstract

The editors of *Chinese Medicine* would like to thank all our reviewers who have contributed to the journal in Volume 9 (2014).

**Rakhi Agarwal**

India

**Catalina Alarcon De La Lastra**

Spain

**Nicolino Ambrosino**

Italy

**Williams Anderson**

United States of America

**Elif Damla Arisan**

Turkey

**Yoichi Asaoka**

Japan

**Niranjan Awasthi**

United States of America

**Jan Axtner**

Germany

**Huveyda Basaga**

Turkey

**Zhaoxiang Bian**

Hong Kong

**Aldo E. Calogero**

Italy

**Huijuan Cao**

China

**Yihai Cao**

Sweden

**Aileen Chan**

Hong Kong

**Thomas Chan**

Hong Kong

**Chun-Tao Che**

United States of America

**Wei Chen**

China

**Jia-Xu Chen**

China

**Chieh-Fu Chen**

Taiwan

**Weiwei Chen**

China

**Guobing Chen**

United States of America

**Haifeng Chen**

China

**Hubiao Chen**

Hong Kong

**Hongli Chen**

China

**Jin-Jer Chen**

Taiwan

**Sibao Chen**

China

**Shilin Chen**

China

**Xiuping Chen**

Macao

**Yu-Chun Chen**

Taiwan

**Ling Cheng**

China

**Juei-Tang Cheng**

Taiwan

**Kur-Ta Cheng**

Taiwan

**Mei Chong**

Canada

**Hy Chung**

Hong Kong

**Vincent Chung**

Hong Kong

**Elise Cogo**

Canada

**Kieran Cooley**

Canada

**Yi Dang**

Hong Kong

**Robert Domitrovic**

Croatia

**Shailesh Dudhgaonkar**

India

**Peter Eckl**

Austria

**Thomas Efferth**

Germany

**Dina El-Agamy**

Egypt

**Daping Fan**

United States of America

**Yutong Fei**

China

**Yibin Feng**

Hong Kong

**Arthur Sa Ferreira**

Brazil

**Fu Fuyou**

United States of America

**Krishnamoorthy Ganesan**

India

**Ting Gao**

China

**Sizhi Paul Gao**

United States of America

**Ilko Getov**

Bulgaria

**Peter Gerard Gibson**

Australia

**Shannon Glaser**

United States of America

**William Goggins**

Hong Kong

**Adel Gomaa**

Egypt

**Yuewen Gong**

Canada

**Sheila Greenfield**

United Kingdom

**Fulan Guan**

United States of America

**Xinfeng Guo**

China

**Young Gwak**

South Korea

**Jianping Han**

China

**Simon Han**

Hong Kong

**Biao He**

United States of America

**Chengwei He**

Macao

**Shanshan He**

United States of America

**Morag Heirs**

United Kingdom

**Suzanne Hendrich**

United States of America

**Janice Yuen-Shan Ho**

Hong Kong

**Hongtao Jin**

China

**Wen-Chi Hou**

Taiwan

**Hao Hu**

Macao

**Kai Hu**

Germany

**Linfang Huang**

China

**Shuang Huang**

United States of America

**Xi Huang**

China

**Lucy Hwang**

Taiwan

**Guang Ji**

China

**Han Jia**

China

**Zhi-Hong Jiang**

Macao

**Jinxin Liu**

China

**Hitoshi Kagaya**

Japan

**Henry J Kaplan**

United States of America

**Hyung Sik Kim**

South Korea

**Kam Ming Ko**

Hong Kong

**Yeng Yeng Kong**

Hong Kong

**Katarzyna Kowalska**

Poland

**Hoi-Hin Kwok**

Hong Kong

**Jung Nien Lai**

Taiwan

**Kristina Lamas**

Sweden

**Kajsa Landgren**

Sweden

**Bombi Lee**

South Korea

**David Lee**

United States of America

**Myeong Soo Lee**

South Korea

**Chung Hang Leung**

Macao

**Siu-wai Leung**

Macao

**Jie Li**

United States of America

**Jian Li**

China

**Xican Li**

China

**Lei Li**

China

**Peng Li**

United States of America

**Peng Li**

Macao

**Shao Li**

China

**Song-Lin Li**

China

**Yaolan Li**

China

**Xing-Tai Li**

China

**Henry Liang**

Australia

**Fanrong Liang**

China

**Zhitao Liang**

Hong Kong

**Dong Wook Lim**

South Korea

**Houwen Lin**

China

**Zhixiu Lin**

Hong Kong

**Shuibin Lin**

United States of America

**Haibo Liu**

China

**Feng-Bin Liu**

China

**Ping Liu**

China

**Yang Liu**

United States of America

**Konstantinos Loupasakis**

United States of America

**Aiping Lu**

Hong Kong

**Lin Lu**

China

**Guanghua Lu**

China

**Zhongqiu Lu**

China

**Jacqueline Lui**

Hong Kong

**Yangchao Luo**

United States of America

**Dik Lung Ma**

Hong Kong

**Xiaocui Ma**

United States of America

**Nripendranath Mandal**

India

**Paul Mcnamee**

United Kingdom

**Jin Mou**

Canada

**Kunal Mukhopadhyay**

India

**Takayuki Nakagomi**

Japan

**Sai Ming Ngai**

Hong Kong

**Taketo Okada**

Japan

**Marina Okoshi**

Brazil

**Aihua Ou**

China

**Mathias Oulé**

Canada

**Roman Pfeifer**

Germany

**Pawinee Piyachaturawt**

Thailand

**Yuyue Qin**

China

**Eamonn Quigley**

Ireland

**Sathishkumar Ramalingam**

India

**José Carlos Rebuglio Vellosa**

Brazil

**Jian-Guo Ren**

United States of America

**See Voon Seow**

Singapore

**Masoomeh Shams-Ghahfarokhi**

Iran

**Hongcai Shang**

China

**Xueguang Shao**

China

**Pang-Chui Shaw**

Hong Kong

**Johannah Shergis**

Australia

**Shi-Bing Su**

China

**Sammy Siu**

Hong Kong

**Jingyuan Song**

China

**Fengrui Song**

China

**Kevin Spelman**

United States of America

**Hutcha Sriplung**

Thailand

**Rowan Stringer**

Switzerland

**Hermann Stuppner**

Austria

**Huanxing Su**

Macao

**Nikolaus Sucher**

United States of America

**Sekhar Surapaneni**

United States of America

**Stephen Cho Wing Sze**

Hong Kong

**Erna Sziksz**

Hungary

**Lida Tang**

China

**Wei Tang**

Japan

**Wenfu Tang**

China

**Jinzhou Tian**

China

**Karl WK Tsim**

Hong Kong

**Ruchan Uslu**

Turkey

**Kaviyarasan Venkatesan**

India

**Sivarama Prasad Vinjamury**

United States of America

**Chao Wan**

Hong Kong

**Jian-Bo Wan**

Macao

**Neng Wang**

Hong Kong

**Zhao Wang**

United States of America

**Ying Wang**

Macao

**Jinn-Chyi Wang**

Taiwan

**Jung-Der Wang**

Taiwan

**Di Wang**

China

**Ping Wang**

United States of America

**Nan Wang**

United States of America

**Pei Wang**

China

**Shu-Ming Wang**

United States of America

**Tianfang Wang**

China

**Zhijun Wang**

China

**Zhiyu Wang**

China

**Jon Wardle**

Australia

**Natthida Weerapreeyakul**

Thailand

**Zehuai Wen**

China

**Martin Wiemers**

Germany

**John Windsor**

New Zealand

**Vincent Kam Wai Wong**

Macao

**Ricky NS Wong**

Hong Kong

**Vivian Wong**

Hong Kong

**Wendy Wong**

Hong Kong

**Darong Wu**

China

**Taixiang Wu**

China

**Liwei Xie**

United States of America

**Pei-Shan Xie**

China

**Jaya Parkash Yadav**

India

**Yukihiro Yamaguchi**

United States of America

**Ru Yan**

Macao

**Chae Ha Yang**

South Korea

**Jie Yang**

China

**Rong-Sen Yang**

Taiwan

**Xianwen Yang**

China

**Meihua Yang**

China

**Yi-Ning Yang**

China

**Fred CT Yen**

Taiwan

**Hin Wing Yeung**

Macao

**Yi Tao**

Hong Kong

**Guohua Yin**

China

**Sheng Yin**

China

**Yongliang Jia**

Macao

**QW Yu**

China

**Hua Yu**

China

**Ti-Fei Yuan**

China

**Jianbo Yue**

China

**Patrick Yue**

Hong Kong

**Sze Chung Yuen**

Macao

**Hengchang Zang**

China

**Christopher Zaslawski**

Australia

**Qingwen Zhang**

Macao

**Suzhi Zhang**

China

**Yanbo Zhang**

Hong Kong

**Xiaojun Zhang**

China

**Zunjian Zhang**

China

**Guo-Qing Zheng**

China

**Hai-Meng Zhou**

China

**Guo-Yuan Zhu**

China

**Hongmei Zhu**

Macao

**Lixin Zhu**

United States of America

**Yuri Zhuravlev**

Russia

**Xiaolin Zi**

United States of America

**Jian-Ping Zuo**

China

